# The emergence of mesencephalic trigeminal neurons

**DOI:** 10.1186/s13064-017-0088-z

**Published:** 2017-06-21

**Authors:** Marcela Lipovsek, Julia Ledderose, Thomas Butts, Tanguy Lafont, Clemens Kiecker, Andrea Wizenmann, Anthony Graham

**Affiliations:** 1 Centre for Developmental Neurobiology, Kings College London, London, SE1 1UL UK; 2 Institute of Clinical Anatomy and Cell Analysis, Department of Anatomy, University of Tübingen, Oesterbergstrasse 3, 72074 Tuebingen, Germany; 3 Universitätsmedizin Berlin, NeuroCure - Institute of Biochemistry, ChariteCrossOver, Virchowweg, 610117 Berlin, Germany; 4 School of Life Sciences, University of Liverpool, Liverpool, L69 3BX UK

**Keywords:** Mesencephalic trigeminal nucleus, MTN, MesV, Jaw proprioception, Midbrain, FGF, WNT BMP

## Abstract

**Background:**

The cells of the mesencephalic trigeminal nucleus (MTN) are the proprioceptive sensory neurons that innervate the jaw closing muscles. These cells differentiate close to the two key signalling centres that influence the dorsal midbrain, the isthmus, which mediates its effects via FGF and WNT signalling and the roof plate, which is a major source of BMP signalling as well as WNT signalling.

**Methods:**

In this study, we have set out to analyse the importance of FGF, WNT and BMP signalling for the development of the MTN. We have employed pharmacological inhibitors of these pathways in explant cultures as well as utilising the electroporation of inhibitory constructs in vivo in the chick embryo.

**Results:**

We find that interfering with either FGF or WNT signalling has pronounced effects on MTN development whilst abrogation of BMP signalling has no effect. We show that treatment of explants with either FGF or WNT antagonists results in the generation of fewer MTN neurons and affects MTN axon extension and that inhibition of both these pathways has an additive effect. To complement these studies, we have used in vivo electroporation to inhibit BMP, FGF and WNT signalling within dorsal midbrain cells prior to, and during, their differentiation as MTN neurons. Again, we find that inhibition of BMP signalling has no effect on the development of MTN neurons. We additionally find that cells electroporated with inhibitory constructs for either FGF or WNT signalling can differentiate as MTN neurons suggesting that these pathways are not required cell intrinsically for the emergence of these neurons. Indeed, we also show that explants of dorsal mesencephalon lacking both the isthmus and roof plate can generate MTN neurons. However, we did find that inhibiting FGF or WNT signalling had consequences for MTN differentiation.

**Conclusions:**

Our results suggest that the emergence of MTN neurons is an intrinsic property of the dorsal mesencephalon of gnathostomes, and that this population undergoes expansion, and maturation, along with the rest of the dorsal midbrain under the influence of FGF and WNT signalling.

## Background

The routes through which cranial primary sensory neurons are generated are complex. In contrast to the trunk, where sensory neurons are formed exclusively by neural crest cells, those in the head have disparate embryonic origins. The majority of the cranial sensory neurons are derived from the neurogenic placodes, which are transient focal thickenings of the cranial ectoderm [1–3]. This includes all of those contributing to the epibranchial ganglia: the geniculate, petrosal and nodose, the cells of the vestibuloacoustic ganglion, and many of the neurons of the trigeminal ganglion, and in some vertebrate clades neurons associated with the spiracular/paratympanic organ [4]. Additionally, there are neural crest derived sensory neurons in the head and these will form the proximal ganglia of the IXth and Xth nerves, the Superior and Jugular, as well as contributing to the trigeminal ganglion [1, 3, 5]. Finally, there is one last significant population of sensory neurons in the head which do not lie in a peripheral ganglion but which are situated within the central nervous system (CNS), the cells of the Mesencephalic Trigeminal Nucleus (MTN), (also sometimes abbreviated as MesV), which relay information about the position of the jaw [6, 7].

We know much about how neural crest derived sensory neurons are formed [8, 9] and, over the last two decades, we have developed an increasing understanding of how placodally derived sensory neurons arise [10]. However, we have very little understanding of how the MTN forms, despite the importance of these cells; they are the proprioceptive neurons that innervate the jaw closing muscles and they thus play a central role in coordinating biting and mastication [11, 12]. Furthermore, the evolution of this population is generally believed to have been concomitant with the evolution of jawed vertebrates, the gnathostomes, and to have facilitated that transition [13, 14]. The appearance of jaws necessitated the emergence of novel sensory systems to co-ordinate jaw movement and this need was met by the MTN.

MTN neurons emerge during comparatively early periods, and these are the first born neurons of the mesencephalon in amniotes. They are generated either side of the dorsal midline forming first posteriorly close to the isthmus but then later across the anteroposterior extent of the midbrain [13, 15]. Indeed, the production of these cells occurs over a protracted period, between stages (HH) 14 and 25 in chick [7, 13, 16]. MTN cells differentiate as unipolar neurons that then project axons away from the dorsal midline ventrally towards the sulcus limitans, at which point they make a caudal turn and contribute to the forming lateral longitudinal fasciculus (LLF) [7]. They further extend their axons along this tract crossing the isthmus to enter the hindbrain before their axons bifurcate and one branch exits through rhombomere two and then projects along the mandibular ramus of the trigeminal nerve towards the mandibular arch.

Importantly, there has been controversy over the embryonic origin of these neurons. It was proposed that these cells are neural crest derived [17] and as such would share a common embryonic origin with other proprioceptive sensory neurons such as those found in the dorsal root ganglia at limb levels. However, other studies failed to find support for this view and it was argued that MTN neurons have a CNS origin [13]. More recently genetic fate-mapping in mice has provided definitive proof for this. MTN cells are part of the Wnt3a lineage which is restricted to cells derived from the dorsal midline of the midbrain and does not include neural crest cells [18]. Given that the embryonic origin of these neurons has now been resolved, it is important that we gain an understanding of how these cells are generated.

As with other regions of the developing CNS, cell type specification within the mesencephalon is likely to involve positional cues emanating from signalling centres, which pattern the anteroposterior (AP) and dorsoventral (DV) axes [19, 20]. In the case of the MTN, these cues would include signals from the isthmus for the AP axis, FGF and WNT, and from the dorsal midline for the DV axis, primarily BMP, but also WNT signalling [21]. However, from the few studies that have been reported to date, it is far from clear how, or if, isthmic and dorsal signals act to specify MTN neurons and what the actual roles of these signalling pathways are during the development of this population. Thus, while a previous study of ours found evidence to suggest that FGF signalling from the isthmus promotes MTN formation in chick [13] a more recent study in zebrafish found that FGF activity had a negative effect whilst WNT signalling had a positive effect on MTN differentiation [22]. Other work has shown that dorsal midbrain development involves signals from the roof plate and that positional identity is set at periods just after the onset of MTN formation [23]. However, it is unclear how the generation of these cells fits with this process and whether or not it is impacted by BMP signalling.

In this study, we have set out to analyse the importance of signals derived from the isthmus and dorsal midline for the development of the MTN. We find that, in cultured explants, interfering with either FGF or WNT signalling results in the generation of fewer MTN neurons, and that these pathways have additive effects. However, this population was still present. Contrastingly, abrogation of BMP signalling has no effect. We also find that inhibition of FGF and WNT signalling affects MTN neuronal axonal extension. To complement these studies, we have used electroporation to inhibit BMP, FGF and WNT signalling within dorsal midbrain cells in vivo prior to, and during, their differentiation as MTN neurons. We found that in all cases cells electroporated with any of these inhibitory constructs can differentiate as MTN neurons suggesting that none of these pathways are required cell intrinsically for the emergence of these neurons. Again, we find that inhibition of BMP signalling has no effect on the development of MTN neurons, while inhibition of FGF or WNT signalling has consequences for MTN differentiation. Double electroporation to inhibit both FGF and WNT signalling suggests that these pathways are additive. Finally, we conducted a series of explant studies to determine if the isthmus or the dorsal midline are at all required for the emergence of MTN neurons at stages after the specification of AP and DV identities. We find that MTN neurons are still generated in cultured explanted dorsal midbrains from stage 12 embryos without the isthmus and roof plate. Thus, although MTN neurons are born close to both the isthmus and the roof plate the emergence of these neurons per se does not directly involve ongoing signalling from these structures. Here, we provide evidence suggesting that the generation of MTN neurons is an intrinsic property of the dorsal mesencephalon, with these being the first cohort of neurons to be born here. This population then undergoes expansion along with the rest of the dorsal midbrain under the influence of FGF and WNT signalling. Furthermore, we uncover an additional role for FGF and WNT signalling in supporting extension of MTN axons.

## Methods

### Explant cultures

Fertilised hen’s eggs were incubated at 38 °C until HH stage 11 or stage 13 [24]. Embryos were dissected and extra-embryonic tissue removed. For the pharmacological manipulation of signalling pathways, embryos were cut anterior to the midbrain/diencephalon border and posterior of the otic vesicle. For the midbrain tissue explant cultures, the midbrain region was first dissected and the isthmus or roof plate regions removed using fine tungsten needles. Explant pieces were embedded in type I collagen (Roche), dorsal side up and cultured overnight, at 37 °C in F12 medium (Sigma) supplemented with 10% fetal calf serum (Life Technologies) and penicillin/streptomycin (Sigma). All drugs were dissolved in DMSO to the following stock concentrations: dorsomorphin, 5 mM; SU5402, 34 mM; IWP-2; 1 mM. Stock solutions were stored at −20 °C. Drugs, or equal volume of DMSO (1-9ul /ml depending on the corresponding concertation of the drug) for the controls, were added to the culture medium. Explants were fixed in formaldehyde for subsequent immunostaining.

### In ovo electroporations

Fertilised hen’s eggs were incubated at 38 °C. Eggs were windowed using sharp surgical scissors. The midbrain neural tube was injected with ~100–200 nl of the corresponding plasmid DNA at a concentration of 2 μg/μl. Plasmid constructs used were: CAGGS-GFP, pCAβ-Smad6-IRES-GFP [25], pCAβ-dnFGFR1-IRES-GFP [26] and pCAβ-GSK3-IRES-GFP (this construct was generated by inserting full-length human GSK3 into the pCAβ-IRES-eGFPm5 plasmid) [27]. Three 20 ms/10 V square waveform electrical pulses were passed between electrodes placed on either side of the midbrain with a slight sideways inclination so as to direct the current on a ventral to dorsal and left to right angle, targeting the dorsal midbrain. Approximately 1 ml of Tyrode’s solution with penicillin/streptomycin was added before the eggs were resealed and incubated for a further day at 38 °C. Embryos were collected at stage 18–20, the midbrain and rostral hindbrain regions dissected and fixed with 4% paraformaldehyde (in phosphate-buffered saline). Wholemount midbrains were either processed for immunostaing or cryosectioning.

### Immunofluorescence

Previously fixed explant cultures, electroporated whole midbrains or cryosections were washed three times 30 min in PBS/1% TritonX-100 (PBSTx) before being washed in a blocking solution of 10% goat serum in PBSTx twice for 1 h at room temperature. The relevant primary antibodies were diluted in blocking solution and the tissues incubated at 4 °C for 4 days. Samples were then rinsed in blocking solution and washed three times for 1 h in blocking solution before adding the secondary antibody diluted in blocking solution, and incubated at 4 °C for a further 2–3 days. The primary antibodies used were mouse anti-Isl1/2 at 1:100 (DSHB 39.4D5 created by Jessell, T.M. / Brenner-Morton, S) kindly supplied by Dr. Ivo Lieberam, KCL) rabbit anti-NFM at 1:500 (Abcam) and rabbit anti-phospho-SMAD1/5/8 at 1:100 (Cell Signalling). Secondary antibodies used were Alexa 633-conjugated goat anti-mouse IgG, and Alexa 568 goat anti-rabbit IgG, both at 1:500 (Molecular Probes).

### Imaging, cell counting and statistical analysis

Images were acquired by laser scanning confocal microscopy (Olympus AX70). Image analysis and processing was performed in ImageJ and Photoshop. The cell counting of MTN cells on explant cultured midbrains was performed on z stack pictures from a dorsal view. Images were thresholded for unbiased detection of ISL1/2+ nuclei. The total number of cells in each explant was counted manually, at the dorsal midbrain region, using a cell counting plugin on imageJ. Absolute numbers were then averaged for each experimental condition and normalised to the corresponding mean control value. Bar graphs show normalised mean ± S.E.M. values. Statistical analysis was performed on GraphPad Prism version 6.00 for Windows (GraphPad Software, La Jolla California USA, www.graphpad.com). Pairwise comparisons against the corresponding DMSO controls were performed using the two-tailed Mann Whitney test. Multiple comparisons were performed using Kruskal-Wallis test with Dunn’s correction.

## Results

MTN neurons are generated close to the dorsal midline and in many jawed vertebrates they first appear in the posterior midbrain, close to the isthmus, before spreading anteriorly [13, 15]. Thus, their formation may be controlled by the isthmus and the dorsal midline, and involve signals secreted by these territories. Much is known about the role of the isthmus in early neural development and it has been shown that FGF and WNT signalling from this structure play a key role in midbrain patterning [19, 28–30]. Contrastingly, comparatively little is known about dorsal patterning in the midbrain. However, it has been shown that dorsalising signals are produced by the roof plate and that dorsal and ventral identities are fixed in the chick from HH stage 15 onwards, which is after the initial production of MTN neurons [23].

We therefore sought to assess the roles of key signalling pathways emanating from the isthmus and dorsal midline in the emergence of MTN cells. To achieve this we used two independent and complementary approaches. We performed pharmacological inhibition of signalling pathways on explant cultures of chick embryos, and additionally, analysed the cell intrinsic role of signalling pathways by expression of inhibitory constructs through *in ovo* electroporation of the embryonic dorsal midbrain.

### Pharmacological inhibition of signalling pathways reveals a role for FGF and WNT, but not BMP, signalling in MTN development

We established an ex vivo system that allowed for the controlled application of pharmacological reagents. Embryos were isolated and cut anteriorly at the mesencephalic/diencephalic junction and posteriorly of the otic vesicle, at the middle of the hindbrain. These pieces were then embedded in collagen and cultured overnight. The explanted tissue comprised the normal environment in which MTN cells are generated. After incubation, the explant cultures displayed normal morphology. We performed these experiments at two developmental stages: stage 13, just before the first postmitotic MTN neurons are produced [13], and DV identity is in the process of being fixed; and stage 11, when DV identity in the midbrain is completely labile [23]. After incubation, we assessed the presence of MTN neurons by immunostaining for ISL1/2 and NFM [13]. These neurons are the first born in the midbrain, and remain the only differentiated neurons in the dorsal midbrain for an extended period of time. Consequently, the analysis of early axonal extensions is straightforward.

**Fig. 1:**
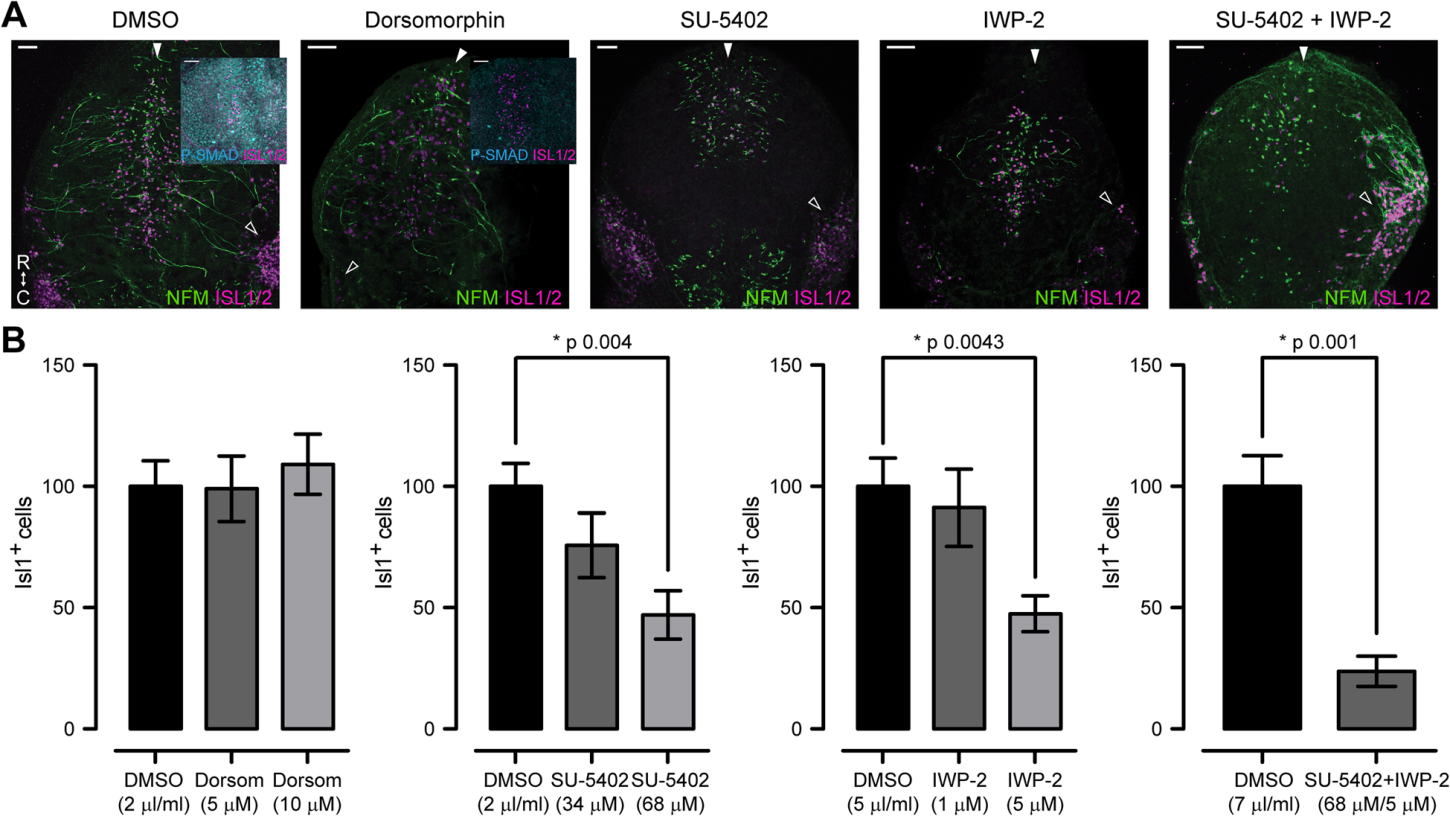
Pharmacological inhibition of signalling pathways on stage 13 explant cultures*.* **a**. Wholemount dorsal view of explant cultures treated with, from left to right, DMSO, dorsomorphin, SU5402, IWP-2 and SU5402 + IWP-2 and immunostained for NFM and ISL1/2. Filled *arrowhead*s show the position of the dorsal midline. Empty arrowheads show the position of the trigeminal placode. R, rostral. C, caudal. Scale bars, 50 μm. Insets, Wholemount dorsal view of explant cultures treated with DMSO or dorsomorphin and immunostained for phospho-SMAD1/5/8 and ISL1/2. Scale bars, 50 μm. **b**. Bar graphs showing ISL1/2+ cell counting on stage 13 explants. Values are mean ± S.E.M. normalised to the mean control value of each experiment. *, significant *p* values for SU5402 and IWP-2 treatments after multiple comparison using Kruskal-Wallis test with Dunn’s correction and for the SU5402 + IWP-2 for pairwise comparison using two-tailed Mann Whitney test. Dorsomorphin: DMSO (*n* = 39), 5 μM (*n* = 19), 10 μM (*n* = 13). SU5402: DMSO (*n* = 33), 34 μM (*n* = 12), 68 μM (*n* = 16). IWP-2: DMSO (*n* = 24), 1 μM (*n* = 10), 5 μM (*n* = 16). SU5402 + IWP-2: DMSO (*n* = 9), 68 μM + 5 μM (*n* = 9). Replicates are from >3independent experiments

To inhibit BMP signalling we used dorsomorphin [31], which selectively inhibits the BMP type I receptors ALK2, ALK3 and ALK6 and thus blocks BMP-mediated SMAD1/5/8 phosphorylation. In explant cultures performed at stage 13, which is just prior to emergence of the first MTN neurons [7, 13], we observed no effect of BMP signalling inhibition. Both the number of MTN cells generated (identified as ISL1/2^+^ cells) and their position within the midbrain showed no differences between control (DMSO treated) explants and dorsomorphin treated explants. Moreover, the initial axon extension also appeared unaffected by dorsomorphin treatment (Fig. 1 a-b). To confirm the effectiveness of the dorsomorphin treatment we performed immunostainings for p-SMAD 1/5/8. While DMSO controls showed strong p-SMAD labelling, this was completely lost in explants treated with dorsomorphin (Fig. 1 a, DMSO and Dorsomorphin panels).

**Fig. 2:**
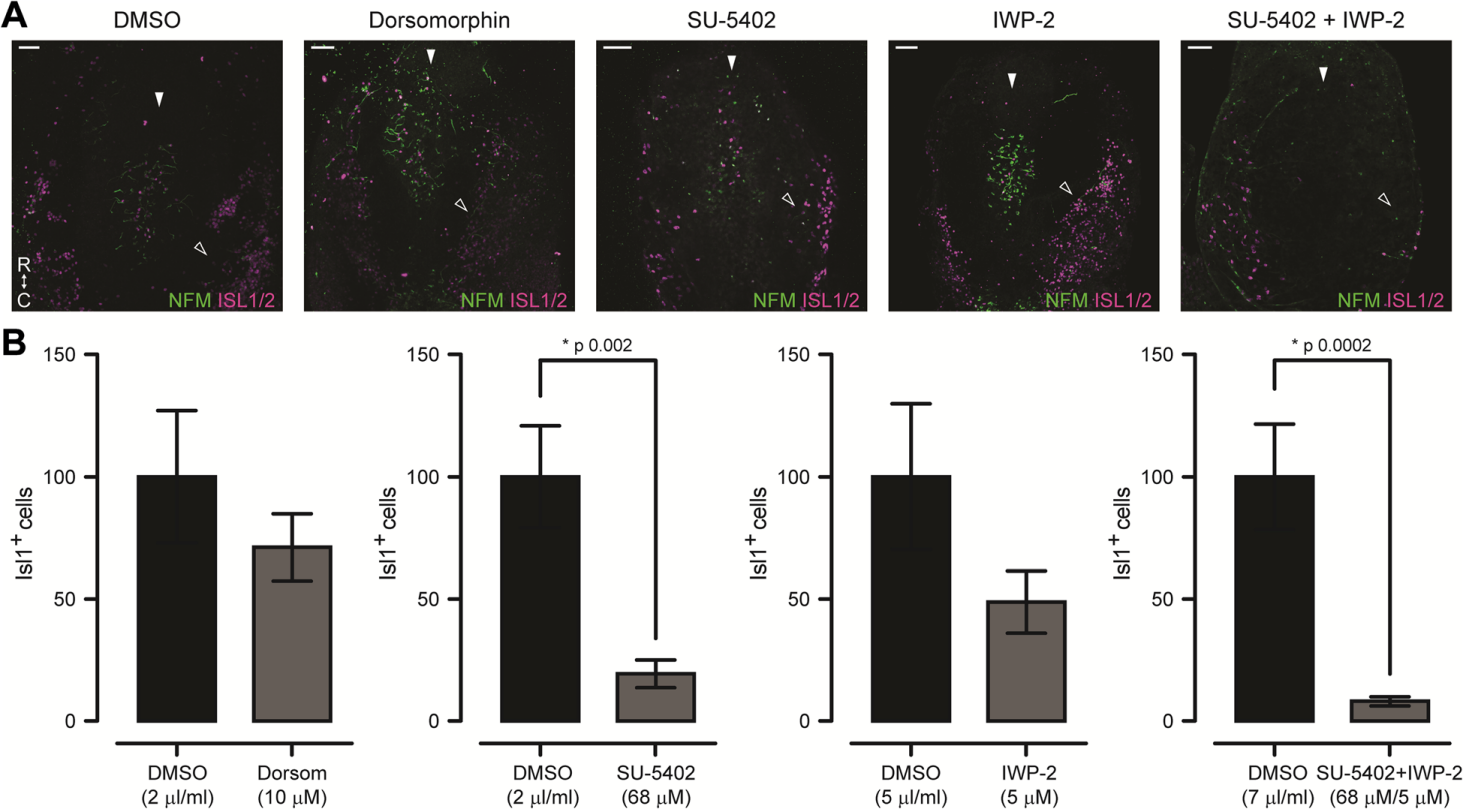
Pharmacological inhibition of signalling pathways on stage 11 explant cultures. **a**. Wholemount dorsal view of explant cultures treated with, from left to right, DMSO, dorsomorphin, SU5402, IWP-2 and SU5402 + IWP-2 and immunostained for NFM and ISL1/2. Filled arrowheads show the position of the dorsal midline. Empty arrowheads show the position of the trigeminal placode. R, rostral. C, caudal. Scale bars, 50 μm. **b**. Bar graphs showing ISL1/2+ cell counting on stage 13 explants. Values are mean ± S.E.M. normalised to the mean control value of each experiment. *, significant *p* values for pairwise comparisons using two-tailed Mann Whitney test. Dorsomorphin: DMSO (*n* = 9), 10 μM (*n* = 12). SU5402: DMSO (*n* = 12), 68 μM (*n* = 9). IWP-2: DMSO (*n* = 9), 5 μM (*n* = 14). SU5402 + IWP-2: DMSO (*n* = 9), 68 μM + 5 μM (*n* = 7). Replicates are from >3 independent experiments

In explant cultures performed at stage 11, we also observed no effects of blocking BMP signalling on MTN development. Both the number of MTN cells generated and their position within the midbrain were indistinguishable from DMSO treated controls (Fig. 2a-b). These results suggest that MTN neuronal development is not under the control of BMP signalling.

To study the effects of FGF signalling from the isthmus on MTN neuronal development, we treated explant cultures with SU5402, which selectively blocks FGFRs by inhibiting their kinase activity [32]. Treatment of stage 13 explant cultures with SU5402 resulted in a significant dose dependent reduction in the number of MTN cells produced, when compared with DMSO treated controls; at the highest concentration of SU5402 tested we observed a 50% reduction in the number of ISL1/2^+^ cells produced. However, we observed no effect on the position of ISL1/2^+^ cells within the midbrain. Yet, axon extension was greatly impaired (Fig. 1a-b).

To further evaluate whether any FGF dependent specification of MTN neurons occurred prior to this stage, we performed explant culture experiments on stage 11 embryos and treated them with SU5402. Here we observed a greater reduction in the number of MTN neurons produced, with SU5402 treated explants showing on average 20% of the number of ISL1/2^+^ cells when compared to DMSO treated controls (Fig. 2 a-b). However, MTN neurons still differentiated.

In order to analyse the role of WNT signalling in MTN neuronal development, we treated explant cultures with IWP-2, an inhibitor of porcupine, the enzyme responsible for the palmitoylation of WNT proteins, which is essential for their secretion [33]. In stage 13 explants inhibition of WNT signalling caused a 50% reduction in the number of MTN neurons, but did not affect the distribution of MTN neurons within the midbrain. The remaining neurons showed abnormal extension of axons (Fig. 1a-b). In explants performed at stage 11 we also observed a tendency towards a reduction in the number of ISL1/2^+^ neurons, albeit not statistically significant (Fig. 2a-b).

To evaluate whether FGF and WNT signalling interact during the development of MTN neurons, we treated explant cultures with SU5402 and IWP-2, to inhibit both pathways. In explant cultures of both stage 13 or stage 11 embryos, we observed an additive effect on the reduction of MTN neurons produced on treated explants when compared to explants treated with each drug individually and DMSO treated controls (Figs. 1 and 2). Inhibition of both FGF and WNT pathways simultaneously did not affect the position of the MTN neurons within the midbrain; however, it greatly affected axon formation. In spite of the observed reduction in the number of neurons produced and their abnormal axonal projections, postmitotic, ISL1/2+ MTN neurons were still generated in the absence of FGF and WNT signalling.

### Inhibition of BMP, FGF and WNT signalling via expression of inhibitory constructs in vivo

Our analysis of the role of BMP, FGF and WNT signalling pathways using pharmacological inhibitors highlighted the involvement of FGF and WNT signalling pathways in aspects of early MTN differentiation. To complement these systemic inhibitory approaches, and to further clarify the possible ongoing roles of these pathways in MTN development, we used in vivo electroporation of pathway manipulation constructs. This approach allows us to assess the cell intrinsic role of signalling pathways on MTN development and, in particular, their initial differentiation and subsequent axon extension. In all cases embryos were electroporated between stages 10 and 13, which is prior to the fixation of dorsal identity and before the formation of any MTN neurons. The embryos were fixed at stages 18–20, by which point MTN neurons have differentiated and have extended axons ventrally and posteriorly.

**Fig. 3:**
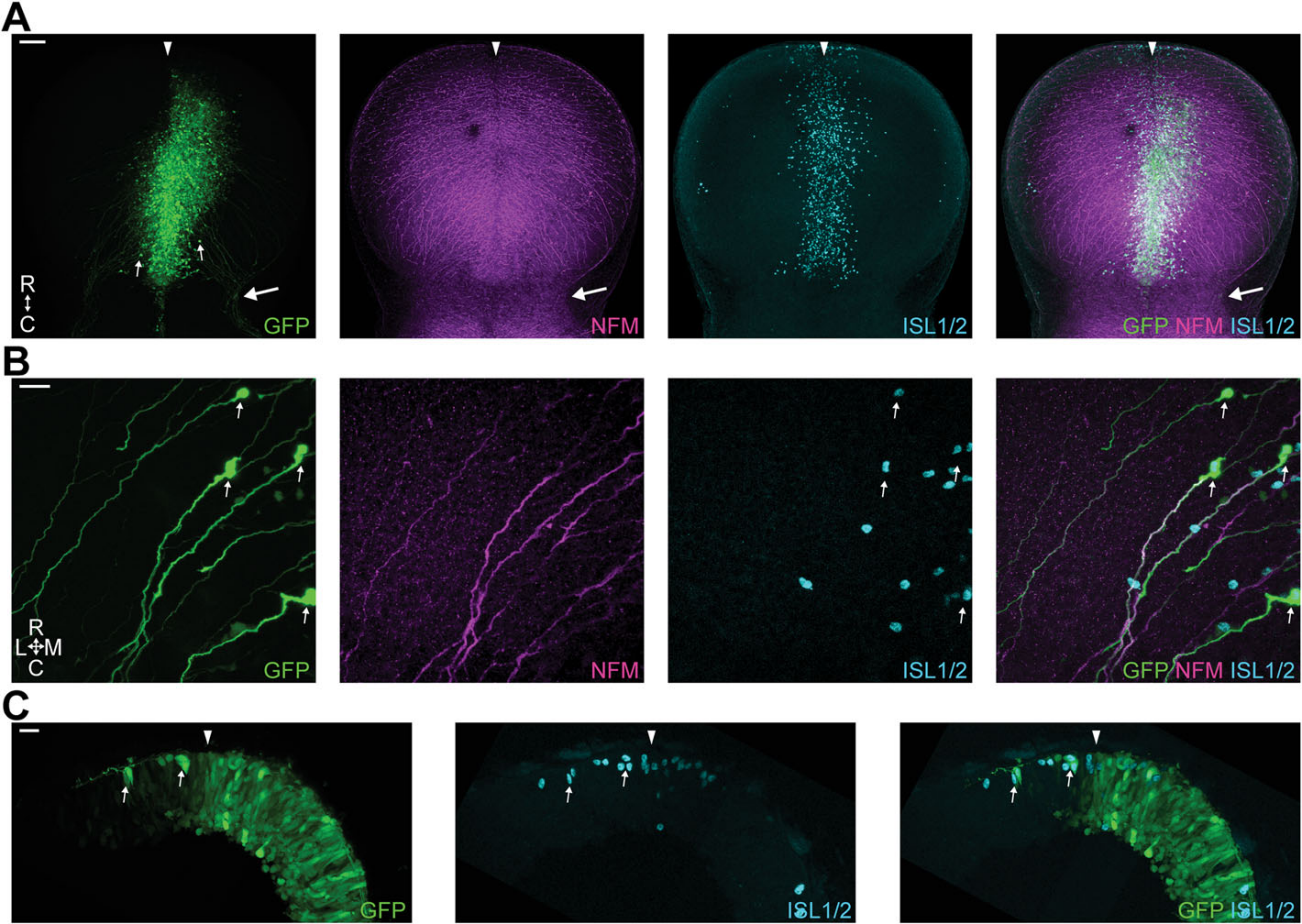
*In ovo* electroporation of the dorsal midbrain. **a**. Representative wholemount dorsal view of a midbrain electroporated with CAGGS-GFP (*n* = 25). *Arrowhead* denotes the position of the dorsal midline. *Thin arrows*, typical MTN neurons. *Big arrow*, right side LLF. Scale bar, 50 μm. **b**. Higher magnification image of GFP-electroporated cells. *Thin arrows*, typical MTN neurons. Scale bar, 20 μm. **c**. Transverse cryosection of a GFP electroporated midbrain. *Arrowhead* denotes the position of the dorsal midline. Thin arrows, typical MTN neurons. Scale bar, 20 μm. R, rostral. C, caudal. M, medial. L, lateral

Figure 3a shows a representative picture of the targeting of the dorsal mesencephalon using a GFP expression construct (the same phenotype was seen in all embryos (*n* = 25)). Labelling was focussed along the dorsal midline (Fig. 3a – filled arrowhead) and the GFP+ cells included both differentiated ISL1+/NFM+ post-mitotic MTN neurons (Fig. 3 a-b, thin arrows) as well as mesencephalic neuroepithelial cells. MTN neurons were also identified by their large somas, lack of dendrites and superficial (pial) location. More importantly, labelling via in vivo electroporation enabled us to scrutinise the axon trajectories of individual cells that carry the construct of interest. A hallmark of MTN neurons is the extension of ventral axonal projections, along the pial surface and with a slight caudal angle. These MTN axons turn sharply caudally at the sulcus limitans where they converge to populate the LLF (Fig. 3a, big arrows) along which they cross the isthmus and head into the hindbrain. Figure 3c shows a representative picture of a transverse cryosection of a GFP electroporated midbrain. On this plane of view the labelling of mesencephalic neuroepithelial cells and differentiated ISL1/2+ neurons (thin arrows) was clearly distinguishable. MTN neurons were identified by their location around the dorsal midline (filled arrowhead) and at the pial surface, ISL1/2 expression, large somas and the characteristic ventrally projecting axon, coursing along the pial surface. These experiments also showed that electroporation per se does not impair MTN development.

### Inhibition of BMP signalling via SMAD6 over-expression has no effect on MTN development

**Fig. 4:**
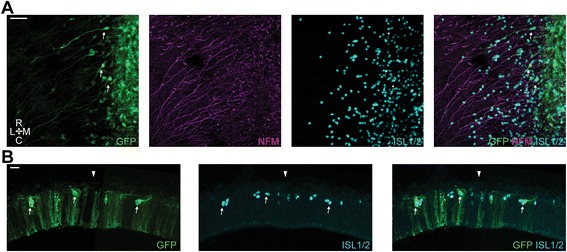
Inhibition of BMP signalling via *in ovo* electroporation of the dorsal midbrain. **a**. Representative high magnification image of Smad6-IRES-GFP electroporated cells. *Thin arrows*, typical MTN neurons. Scale bar, 25 μm (*n* = 8). **b**. Transverse cryosection of a Smad6-IRES-GFP electroporated midbrain. *Arrowhead* denotes the position of the dorsal midline. *Thin arrows*, typical MTN neurons. Scale bar, 20 μm

To assess the requirement for BMP signalling in the generation of MTN neurons and their subsequent early development, chick embryos were electroporated at the dorsal midline with a SMAD6 expression construct, as over-expression of this gene has been shown to block BMP signalling [25]. Electroporations were performed at stages 10–13 and embryos were fixed at stage 18–20 (the same result was seen in all embryos (*n* = 8)). We observed no noticeable effects on midbrain morphology and general distribution of ISL1/2+ cells when comparing SMAD6-IRES-GFP electroporated midbrains against GFP electroporated control midbrains. Figure 4 shows that cells carrying the SMAD6 construct exhibit normal MTN development, which is consistent with our in vitro analyses. GFP+ cells differentiate into postmitotic ISL1/2+ neurons (Fig. 4 a – thin arrows) and were appropriately positioned both along the AP axis and relative to the dorsal midline. Furthermore, these neurons project their axons ventrally towards the LLF in a manner similar to GFP controls. On transverse cryosections we observe that GFP+ MTN neurons were correctly positioned at the pial surface of the neuroepithelium, have the characteristic big somas and extended their axons ventrally coursing right at the pial surface (Fig. 4b – thin arrows). Thus, as with our explant studies, we found no requirement for BMP signalling for the generation and normal early development of MTN neurons.

### Inhibition of FGF signalling via dn-FGFR1 over-expression has no effect on MTN generation but affects axon projection and apicobasal positioning

**Fig. 5:**
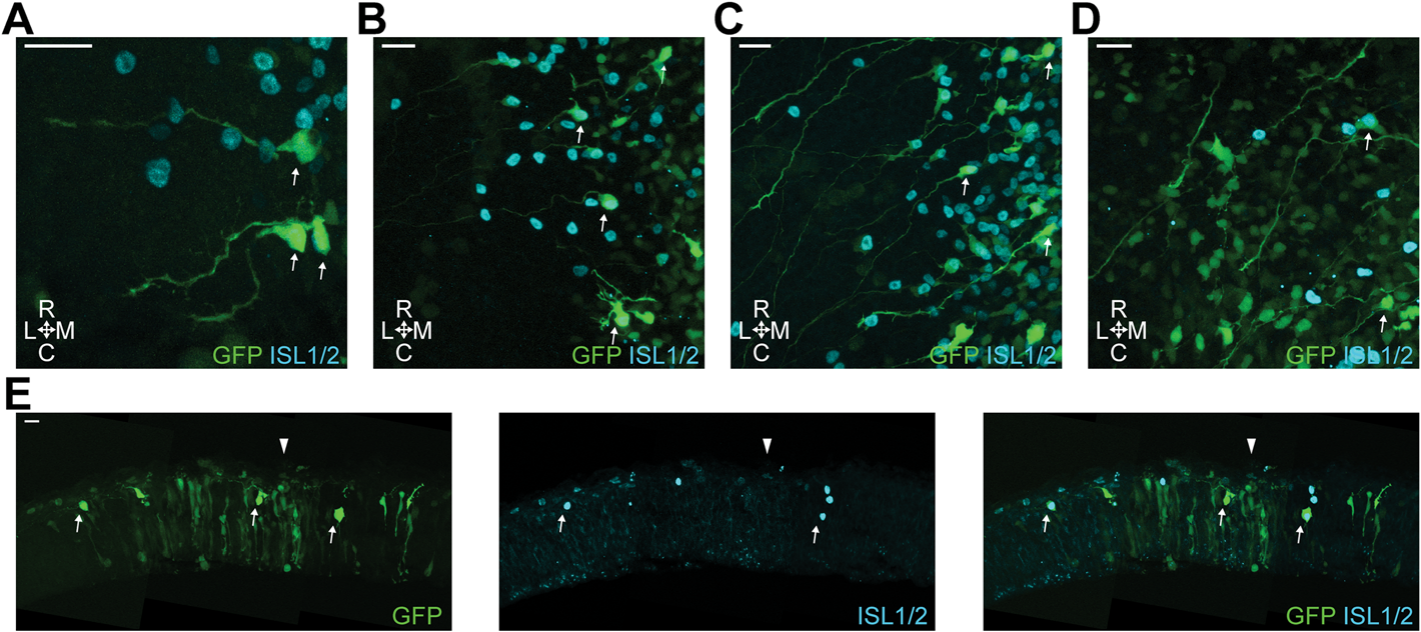
Inhibition of FGF signalling via *in ovo* electroporation of the dorsal midbrain. **a, b, c** and **d**. Representative high magnification images of dnFGFR1-IRES-GFP electroporated cells illustrating the different morphologies observed (*n* = 18). *Thin arrows*, typical dnFGFR1 electroporated MTN neurons. Scale bars, 20 μm. **e**. Transverse cryosection of a dnFGFR1-IRES-GFP electroporated midbrain. *Arrowhead* denotes the position of the dorsal midline. *Thin arrows*, typical dnFGFR1 electroporated MTN neurons. Scale bar, 20 μm

We have previously used electroporation of a dominant negative form of FGFR1 to render neural crest cells insensitive to FGF signalling [26]. We employed the same approach here to assess the requirement for FGF signalling in the differentiation and early development of MTN neurons. Electroporations were performed at stages 10–13 and embryos were fixed at stage 18–20 (the same phenotypes was seen in all embryos (*n* = 18)). We observed no noticeable effects on midbrain morphology and general distribution of ISL1/2+ cells when comparing dnFGFR1-IRES-GFP electroporated midbrains against GFP electroporated control midbrains. Moreover, we found that MTN neurons bearing this construct were produced, as observed by the presence of numerous GFP+/ISL1/2+ cells (Fig. 5 - thin arrows), suggesting that FGF signalling is not required on a cell intrinsic level for their differentiation from dorsal mesencephalic precursors. However, cells carrying the dnFGFR1 construct displayed abnormal axonal projections. Axons often showed a dwindling trajectory with numerous small branchings (Fig. 5a, c and c) or stopped short after a deviated trajectory (Figs. 5a, b and d). In many cases we observed GFP+ and ISL1/2+ cells with a stubby morphology and a high number of very short projections with no apparent general direction (Fig. 5b). Overall, even though we have occasionally observed dnFGFR1 electroporated cells projecting towards the LLF many of these cells showed random projections. Additionally, on transverse cryosections, we observed that the ISL1+/GFP+ MTN cells were often mislocalised and could be found away from the pial surface projecting an axon from this abnormal position (Fig. 5e – thin arrows).

### Inhibition of WNT signalling via GSK3 over-expression has no effect on MTN generation but perturbs axon projection and apicobasal positioning

**Fig. 6:**
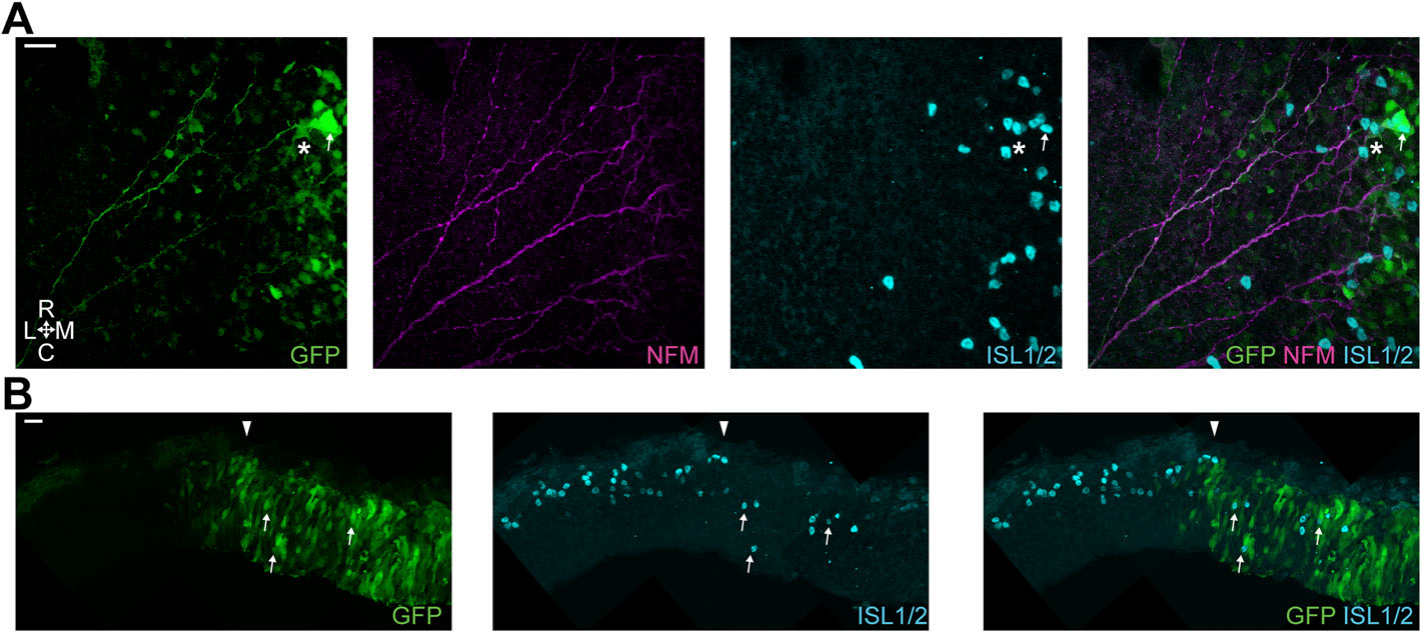
Inhibition of WNT signalling via *in ovo* electroporation of the dorsal midbrain. **a**. Representative high magnification image of GSK3-IRES-GFP electroporated cells (*n* = 19). *Thin arrow*, typical GSK3 electroporated MTN neuron. *Asterisk,* deeper MTN neuron. Scale bar, 20 μm. **b**. Transverse cryosection of a GKS3-IRES-GFP electroporated midbrain. *Arrowhead* denotes the position of the dorsal midline. *Thin arrows*, typical GSK3 electroporated MTN neurons. Scale bar, 20 μm

To assess the requirement for WNT signalling in cells of the dorsal midbrain for early aspects of MTN development, chick embryos were electroporated with a glycogen synthase kinase-3 (GSK3) over-expression construct. GSK3 is a known antagonist of the canonical WNT signalling pathway that forms a complex with, and phosphorylates, the WNT signal transducer β-catenin, thereby marking it for proteasomal degradation. Although GSK3 has many cellular targets (reviewed in [34]), its over-expression in early vertebrate embryos affects the canonical WNT pathway with high specificity [35, 36]. Unlike the WNT inhibitors of the SFRP and DKK families, GSK3 allows blocking of WNT signalling intracellularly, in a cell-autonomous manner. Electroporations were performed at stages 10–13 and embryos were fixed at stage 18–20. All embryos showed the same phenotype (*n* = 19). We observed no noticeable effects on midbrain size and general distribution of ISL1/2+ cells when comparing GSK3-IRES-GFP electroporated midbrains against GFP electroporated control midbrains. We observed the presence of ISL1+/GFP+ MTN neurons (Fig. 6a – thin arrow) indicating that interfering with WNT signalling does not result in a failure of dorsal mesencephalic cells to generate MTN neurons. Also, we noted no difference in the AP position of these neurons, nor their location relative to the dorsal midline. We further found that the axons extended by electroporated neurons were relatively normal; they projected towards the LLF but occasionally exhibited small branches (Fig. 6a – thin arrow). However, transverse cryosections showed that GFP+ MTN neurons were misplaced within the apicobasal axis of the neuroepithelium. Many of the ISL1+/GFP+ cells had not reached the pial surface (Fig. 6b – thin arrows). In line with this observation, we could observe GFP+/ISL1+ cells extending axons ventrally, but positioned one on top of the other (dorsal view of Fig. 6a). While the axon of the cell on top (thin arrow) was most likely following the normal MTN route along the pial surface, the axon of the second cell (asterisk) headed ventrally embedded in the neuroepithelium.

### Combinatorial inhibition of FGF and WNT signalling has no effect on MTN generation but affects axon projection and apicobasal positioning

**Fig. 7:**
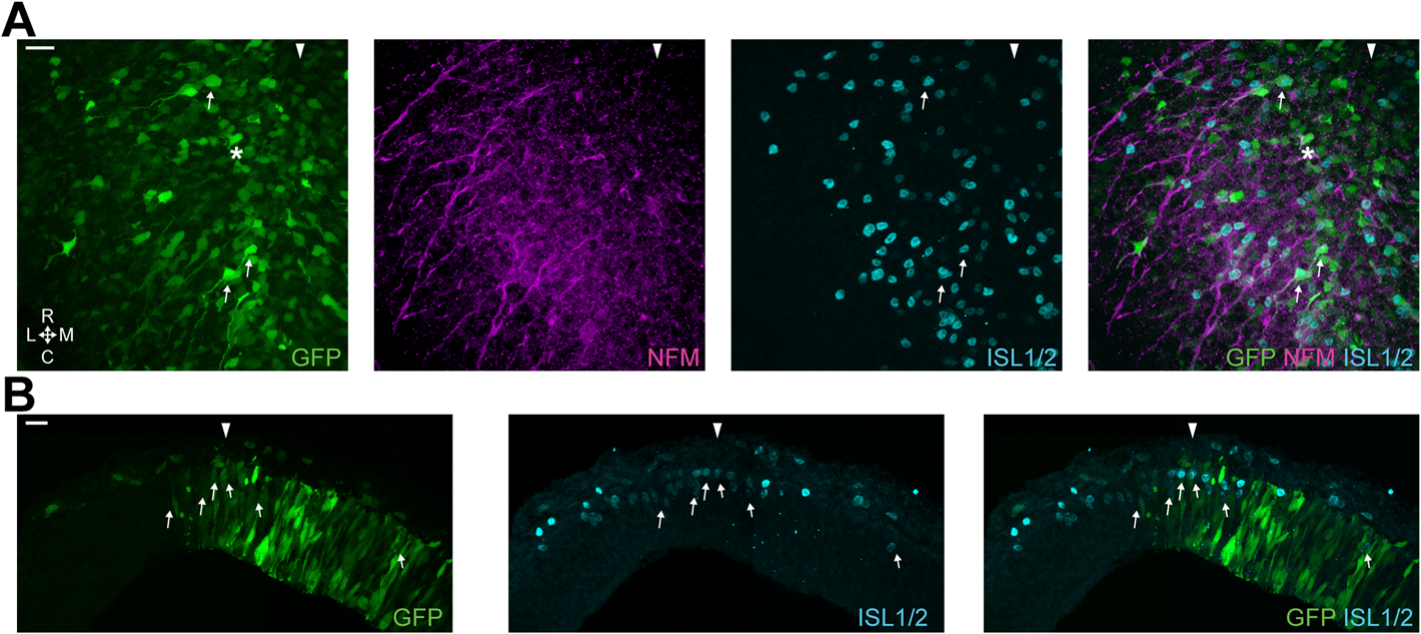
Inhibition of FGF and WNT signalling via *in ovo* electroporation of the dorsal midbrain. **a**. Representative high magnification image of dnFGFR1-IRES-GFP and GSK3-IRES-GFP electroporated cells (*n* = 11). *Thin arrow*, typical dnFGFR1 + GSK3 electroporated MTN neurons. *Asterisk*, Stubby GFP+ cell. *Arrowhead* denotes the position of the dorsal midline. Scale bar, 20 μm. **b**. Transverse cryosection of a dnFGFR1-IRES-GFP and GKS3-IRES-GFP electroporated midbrain. *Arrowhead* denotes the position of the dorsal midline. *Thin arrows*, typical GSK3 electroporated MTN neurons. Scale bar, 20 μm

Our explant studies suggest that FGF and WNT signalling may have an additive effect during MTN differentiation. We therefore sought to analyse how these signalling pathways may affect MTN development in vivo on a cell intrinsic level via a combined electroporation with the dnFGFR1-IRES-GFP and GSK3-IRES-GFP constructs. Electroporations were performed at stages 10–13 and embryos were fixed at stages 18–20. The same phenotype was seen in all embryos (*n* = 11). We observed no noticeable effects on midbrain size and general distribution of ISL1/2+ cells when comparing dnFGFR1-IRES-GFP + GSK3-IRES-GFP electroporated midbrains against GFP electroporated control midbrains, or between electroporated and non electroporated areas of each individual midbrain. Moreover, we found that MTN neurons were generated, as depicted by the numerous GFP+ and ISL1/2+ cells observed (Fig. 7a – thin arrows), and correctly positioned on the AP and DV axes. The axonal phenotypes displayed by the ISL1+/GFP+ cells resembled those associated with cells electroporated with either construct alone (see Figs. 5 and 6). Thus, axons from ISL1+/GFP+ neurons often showed branchings, dwindling trajectories, numerous small branchings and axons coursing underneath other MTN cells (Fig. 7a – thin arrows). Once again we also observed GFP+ cells with a stubby morphology depicting a high number of very short projections with no apparent general direction (Fig. 7a – asterisk). On transverse cryosections we observed many GFP+ and ISL1/2+ cells misplaced within the neuroepithelium, positioned away from the pial surface (Fig. 7b – thin arrows).

### MTN formation does not depend on ongoing isthmus or roof plate signalling

**Fig. 8:**
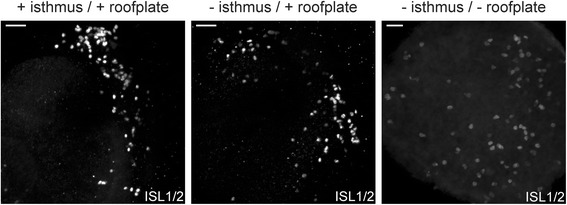
Cultured explants of midbrain neural tube. Representative wholemount views of midbrain explant cultures. Left, whole midbrain with the isthmus (*n* = 6); middle, midbrain without the isthmus (*n* = 22); right midbrain without the isthmus and roof plate (*n* = 38). Immunostained for ISL1/2. Scale bars. 50 μm

MTN neurons emerge close to the isthmus and the dorsal midline, two major signalling centres in the developing midbrain. Consequently, it might be anticipated that these would act to direct the formation of MTN neurons. However, our analysis of the requirement for BMP, FGF and WNT signalling in MTN generation suggests that none of these signals are intrinsically required for the initial generation of these neurons, although FGF and WNT signalling are important for increasing the size of this population and initial extension of axonal projections. We have therefore used explants studies to directly test if these signalling centres, the isthmus and/or the roof plate, are required for the production of MTN neurons. We cultured explants of dorsal midbrain neural tube from embryos at stage 11/12 with and without the isthmus and the roof plate. Explants were embedded in collagen gel and cultured overnight. In all control explants we found that many ISL1/2+ MTN neurons were generated in all explants analysed (*n* = 6/6) (Fig. 8). However, we also found many ISL1/2 neurons in explants cultured without the isthmus (*n* = 22/22) (Fig. 8). Finally, we found that explants lacking both the isthmus and the roof plate also generated ISL1/2+ MTN neurons (*n* = 30/38) (Fig. 8). Overall, our results show that the generation of MTN neurons is intrinsic to dorsal midbrain tissues from stage 10 onwards and does not involve isthmic or roof plate signals.

## Discussion

Mesencephalic trigeminal neurons form in close proximity to the signalling centres of the dorsal midbrain, the isthmus and the roof plate. In this study, we have analysed the importance of key signalling pathways emanating from these organising centres on the development of these cells. These pathways include FGF and WNT signalling, which are involved in mediating isthmic patterning along the AP axis, and BMP signalling which is primarily associated with the dorsal midline and the patterning of dorsal neuronal populations of the developing CNS, although a role for WNT signalling from the dorsal midline is also possible [20, 21]. We inhibited these pathways using pharmacological reagents in cultured explants and via expression of inhibitory constructs of these pathways in vivo. Systemic inhibition of FGF and WNT signalling in explants demonstrated that these pathways impact upon the production of MTN neurons. When inhibited the number of MTN cells is significantly reduced. However, we also find that dorsal midbrain cells rendered insensitive to FGF and WNT signalling still progress to differentiate as MTN neurons. Nonetheless, we show that these signalling pathways are important for aspects of MTN neuronal development. When FGF and WNT signalling are inhibited the early axonal projections are abnormal; they are shorter, exhibit more processes and show more random projection patterns, and MTN neurons are often apicobasally misplaced within the neuroepithelium. Perhaps most surprisingly, we find no evidence for a role for BMP signalling in the generation of these cells or in the early phase of neuronal development, suggesting that the development of the MTN is regulated in a manner different to dorsal neurons elsewhere in the developing neural tube. Finally, our explant data suggest that the generation of MTN neurons is intrinsic to the dorsal midbrain, starting from relatively early stages following the specification of AP and DV identities, and that neither the isthmus nor the roof plate have any direct role in the emergence of these cells although they do play roles in aspects of their early differentiation.

Earlier studies have implicated isthmic signals as playing a role in directing the early development of the MTN in both chick and zebrafish. We previously demonstrated that FGF signalling has a positive role in promoting MTN production in chick, but the role of WNT signalling was unclear [13]. We show here, however, that inhibition of both FGF and WNT signalling in explants results in a reduction in the number of MTN neurons. This contrasts with a recent study in zebrafish which also found roles for both FGF and WNT isthmic signalling in MTN specification [22]. In this species, loss of FGF signalling results in an increase in the number of MTN neurons while loss of WNT signalling results in a decrease; additionally reduced FGF signalling in zebrafish results in the differentiation of MTN neurons closer to the isthmus. This suggests that the development of the MTN is differentially controlled by the isthmus in zebrafish versus chick. However, it should be noted that the position of the initial differentiation of MTN neurons differs between these two species. In zebrafish MTN neurons first differentiate in the anterior mesencephalon and not close to isthmus as seen in chick [22]. Studies of MTN development in a number of other species suggest that the situation in zebrafish represents a derived condition while that found in chick is more representative of a plesiomorphic state; in both chondrichthyans (catshark) and mammals (mouse) MTNs also differentiate close to the isthmus [15].

The results presented here show that, while systemic inhibition of FGF or WNT signalling from stages just prior to the onset of neuronal differentiation affects the number of MTN cells being produced, dorsal midbrain cells insensitive to both of these pathways can differentiate as MTN neurons. Thus, both FGF and WNT signalling are likely to affect MTN development via their more general roles in the growth of the midbrain [37], as well as promoting proliferation and cell survival [38–40]. Indeed, if these pathways are inhibited at earlier stages more drastic effects will result in a failure of general midbrain development and, secondarily, in the loss of MTN neurons. However, on a cell intrinsic level these pathways are not immediately required for MTN differentiation. Cells of the dorsal mesencephalon electroporated with inhibitory constructs for FGF and/or WNT signalling will progress to differentiate as MTN neurons. Indeed, we provide direct support for this assertion from our explant studies which show that early dorsal midbrain tissue will go on to generate MTN neurons without isthmic or roof plate cues. This result is in keeping with an earlier analysis of the wnt1 mutant mouse which completely lacks the midbrain and rhombomere 1 but which nonetheless generates dorsal neurons with caudal projections, although the identity of these is ill defined [30].

Yet, our study shows that, while MTN cells are still produced, inhibition of FGF and WNT signalling did affect their development and resulted in a failure to properly establish initial axonal projections. The axons themselves were reduced and rather than projecting towards the presumptive LLF the projections were misdirected. We also found that inhibiting these pathways in vivo resulted in MTN neurons being misplaced apicobasally within the neuroepithelium. Thus, the MTN may represent an example of a post-mitotic neuronal cell type responding to FGF and WNT mediated projection and maturation cues. Notably, the projections of trochlear neurons, which are largely a rhombomere 1 derivative, are also directed by isthmic FGF signalling [41] and WNT signalling has been implicated in the guidance of a number of different neural populations [42]. While the effects we observed here are most likely cell autonomous, it is possible that electroporated cells could affect their non-electroporated neighbours and this would be worthwhile investigating.

Perhaps the most surprising finding from our study is that BMP signalling has no role in the early development of the MTN. In the spinal cord BMP signalling plays a key role in the generation and patterning of different dorsal interneuron populations [20] suggesting that BMP signalling may also be important for the specification of MTN neurons. These cells are the first born neuronal population of the dorsal mesencephalon, appearing either side of the midline form stage 14 onwards, and at this time the dorsal midline expresses BMPs, in particular GDF7. However, here we show that the generation of MTN neurons occurs independently of such a signal. This suggests that the specification of these neurons takes place outside the framework that underpins the formation of dorsal neuronal cell types in other regions of the developing CNS. Further support for the view that BMP signalling plays little role in early dorsal midbrain patterning arises from the observation that the manipulation of BMPR1B signalling has no effect upon dorsal midbrain specification [43]. In the present study, we further show that the generation of MTN neurons occurs independently of any roof plate influence, even at stages before DV identity in the midbrain is determined. A possible explanation for the absence of a role for the roof plate or BMP signalling in the specification of MTN neurons may lie in the fact that unlike in the spinal cord, there are no cell fate choices to be made in the early midbrain. Thus, while in the dorsal spinal cord BMP signalling specifies the generation of dI1–3 neurons, the only cell type generated in the dorsal midbrain between stages 14 and 23 in the chick are the MTN cells.

Our results therefore suggest that the specification of neurons of the MTN requires only the following three parameters – they are mesencephalic in origin, they are dorsal and they are the first born neurons in this territory. We further find that although the signalling centres of the midbrain, the isthmus and roof plate have no direct inputs into the generation of MTN neurons, they exert profound effects on the MTN, acting to expand this population and to direct their initial axon trajectories.

Finally, the enigmatic location of MTN neurons within the CNS rather than there being a corresponding group of neurons within the trigeminal ganglia is still an unresolved question. There may be two answers to this and both may relate to the evolutionary origins of the MTN. Firstly, the presence of primary sensory neurons within the dorsal CNS is an ancestral feature of chordates: Rohon-Beard cells are present in amphioxus and in larval anamniotes [44] and furthermore, lampreys possess a group of dorsal sensory medullary cells, primary medullary and spinal nucleus of the trigeminal nerve (PMSV) neurons, which display many similarities to Rohon-Beard cells but which persist to adulthood [14]. Thus, we propose that, in the gnathostome lineage, as the jaw apparatus and its associated musculature were assembled, a novel group of sensory neurons emerged in dorsal CNS in proximity to the trigeminal system. The emergence of MTN neurons within dorsal rhombomere 1 may have been precluded by the allocation of this territory to the generation of cerebellar structures and consequently the dorsal midbrain would be the closest CNS area to the trigeminal system with the potential to generate these sensory neurons. Furthermore a midbrain origin would allow MTN neurons to integrate visual information and modulate the position of the jaw in response to that [45]. Secondly, within the cranial sensory ganglia modalities tend to be clearly segregated; for example gustation is associated with the epibranchials, hearing and balance with the vestibuloaccoustic and general somatosensation with the trigeminal placodes [46]. As such the generation of a novel group of proprioceptive neurons within the existing trigeminal ganglia may not have been a possibility and thus dorsal sensory cells of the CNS were co-opted to supply the proprioceptive innervation of the jaw closing muscles.

## Conclusions

The mesencephalic trigeminal nucleus (MTN) is an important and enigmatic population forming close to two key signalling centres that impact upon the development of the dorsal midbrain, the isthmus which mediates its effects via FGF and WNT signalling and the roof plate which is a major source of BMP as well as WNT signalling. We show that interfering with either FGF or WNT signalling has pronounced effects on MTN development whilst abrogation of BMP signalling has no effect; inhibition of either FGF or WNT signalling results in the generation of fewer MTN neurons and axonal defects. However, we find cells refractory to either FGF or WNT signalling can differentiate as MTN neurons suggesting that these pathways are not required cell intrinsically for their emergence. We also show that explants of the dorsal mesencephalon lacking the isthmus and roof plate can generate MTN neurons. Our results suggest that the emergence of MTN neurons is an intrinsic property of the dorsal mesencephalon of gnathostomes, and that this population undergoes expansion, and maturation, along with the rest of the dorsal midbrain under the influence of FGF and WNT signalling.

## Abbreviations

LLF: Lateral longitudinal fasciculus
MTN or MesV: Mesenecephalic trigeminal nucleus
